# Spinopelvic Realignment and Clinical Outcomes After Surgical Management of Adult Degenerative Lumbar Deformity: A Multicenter Retrospective Cohort Study

**DOI:** 10.3390/jcm15135280

**Published:** 2026-07-06

**Authors:** Sanubar Nazarli, Teoman Bircan, Doğan Güçlühan Güçlü, Altay Sencer

**Affiliations:** 1 Department of Neurosurgery, Istanbul Aydın University VM Medical Park Florya Hospital, 34295 Istanbul, Turkey; 2 Faculty of Medicine, Istanbul Aydın University, 34295 Istanbul, Turkey; 3 Department of Neurosurgery, Faculty of Medicine, Istanbul University, 34452 Istanbul, Turkey

**Keywords:** adult degenerative lumbar deformity, spinopelvic realignment, pelvic incidence–lumbar lordosis mismatch, sagittal vertical axis, lumbar lordosis, osteoporosis, revision surgery, patient-reported outcomes

## Abstract

**Background/Objectives:** Adult degenerative lumbar deformity is a heterogeneous condition in which outcome depends on radiographic correction, patient-related risk factors, and surgical burden. This study evaluated spinopelvic realignment, clinical outcomes, complications, and predictors of unfavorable postoperative course after surgical treatment of adult degenerative lumbar deformity. **Methods:** This three-center retrospective cohort study included adult patients who underwent posterior decompression and instrumented fusion, with or without interbody fusion, for adult degenerative lumbar deformity between January 2021 and December 2024. Of 136 screened patients, 113 completed final follow-up and were included in the analysis. The mean follow-up duration was 31.0 ± 12.9 months. Radiographic parameters were assessed preoperatively, immediately postoperatively, and at final follow-up. Patient-reported outcome measures were analyzed using available paired data. Unfavorable postoperative course was defined as persistent or worsened pain with functional limitation, symptomatic mechanical complication, deep infection requiring surgical treatment, or revision/reoperation. **Results:** Surgery produced significant immediate improvement in coronal and sagittal alignment. Cobb angle improved from 29.8 ± 13.1° to 13.7 ± 6.7°, lumbar lordosis increased from 28.8 ± 15.5° to 40.3 ± 16.0°, PI–LL mismatch decreased from 21.7 ± 10.0° to 10.1 ± 11.5°, and SVA decreased from 58.8 ± 31.4 mm to 32.5 ± 36.0 mm. Partial loss of correction was observed at final follow-up, although alignment generally remained improved compared with baseline. ODI improved from 57.8 ± 12.6 to 34.7 ± 8.7 in patients with available paired data. Any postoperative complication occurred in 42.5% (*n* = 48) of patients, revision/reoperation in 23.9% (*n* = 27), and unfavorable postoperative course in 35.4% (*n* = 40). In multivariable analysis, osteoporosis, greater fusion length, and residual immediate postoperative PI–LL mismatch were independently associated with unfavorable postoperative course. **Conclusions:** In this three-center retrospective cohort, surgery for adult degenerative lumbar deformity was associated with significant radiographic correction and meaningful clinical improvement in patients with available paired outcome data. However, the substantial complication and revision/reoperation burden highlights the morbidity of adult degenerative lumbar deformity surgery. Osteoporosis, fusion length, and residual immediate postoperative PI–LL mismatch may help identify patients at higher risk for unfavorable postoperative course.

## 1. Introduction

Adult degenerative lumbar deformity represents a heterogeneous and increasingly common clinical problem in the aging population. This spectrum may include degenerative lumbar scoliosis, lumbar spinal stenosis with deformity, degenerative spondylolisthesis, loss of lumbar lordosis, and sagittal or coronal malalignment. Patients often present with a combination of mechanical back pain, radiculopathy, neurogenic claudication, functional limitation, and impaired health-related quality of life (HRQOL) [1,2,3].

Surgical treatment can provide meaningful improvement in pain, disability, radiographic alignment, and HRQOL in selected patients with adult spinal deformity and degenerative lumbar deformity [4,5,6]. However, these procedures are frequently complex and may require multilevel decompression, long-segment instrumentation, interbody fusion, osteotomy, and spinopelvic realignment. As a result, postoperative complications, mechanical failure, and revision surgery remain major concerns, particularly in older patients with comorbidities, poor bone quality, and long fusion constructs [7,8,9,10].

Sagittal spinopelvic alignment has become central to adult deformity assessment and surgical planning. Positive sagittal balance is associated with worse clinical outcomes, and parameters such as the sagittal vertical axis (SVA), pelvic tilt (PT), and pelvic incidence–lumbar lordosis (PI–LL) mismatch have been shown to correlate with disability and HRQOL [11,12]. These concepts were incorporated into The Scoliosis Research Society (SRS)–Schwab classification, which provides a clinically relevant framework for describing the severity of adult spinal deformities and guiding treatment decisions [13].

Nevertheless, the relationship between radiographic correction and clinical improvement is not always straightforward in adult degenerative lumbar deformity. Unlike more uniform deformity populations, these patients frequently present with overlapping deformities, stenosis, instability, neurological symptoms, pain generators, and variable compensatory capacity. Previous studies have shown that the correlations between spinopelvic parameters and patient-reported outcomes may be modest, particularly in degenerative lumbar scoliosis with concomitant stenosis or in analyses adjusted for patient-related factors [1,3,14]. More recent evidence suggests that postoperative alignment and early spinopelvic correction may be clinically relevant, especially for residual PI–LL mismatch, SVA, T1 pelvic angle (TPA), and lordosis-related global sagittal realignment [3,15,16,17].

At the same time, the optimal correction strategy remains a matter of balance. Residual malalignment may contribute to persistent symptoms and mechanical overload, whereas excessive correction may increase junctional risk, especially in older or osteoporotic patients. This has led to an increasing emphasis on individualized and age-adjusted alignment targets rather than uniform correction thresholds [18,19,20,21]. However, data remain limited regarding how early postoperative residual alignment interacts with patient-related risk factors, bone quality and operative burden to influence the final postoperative course in adult degenerative lumbar deformity. The PI–LL mismatch was selected as the principal postoperative alignment parameter because it reflects the relationship between pelvic morphology and achieved lumbar lordosis, is widely used in adult deformity assessment, and has been linked to disability, mechanical loading, and age-adjusted alignment goals. We hypothesized that residual immediate postoperative PI–LL mismatch, osteoporosis and greater fusion extent would be associated with an unfavorable postoperative course after surgical treatment of adult degenerative lumbar deformity.

The novelty of this study lies in linking this immediate postoperative residual alignment marker with patient-related risk and operative burden in a single three-center adult degenerative lumbar deformity cohort. By focusing on variables available before or immediately after surgery, the analysis aimed to identify risk signals before late mechanical complications or revision surgery become clinically evident.

## 2. Materials and Methods

### 2.1. Study Design and Setting

This multicenter retrospective cohort study was conducted using data from three centers and was prepared in accordance with the Strengthening the Reporting of Observational Studies in Epidemiology (STROBE) statement [22]. Data were collected from three centers where the participating surgeons had practiced during the study period. Consecutive adult patients who underwent surgical treatment for adult degenerative lumbar deformity between January 2021 and December 2024 were screened for eligibility. Because of the retrospective cohort design, no formal a priori sample size calculation was conducted. The sample size was determined by the number of consecutive eligible patients treated during the predefined study period who met the inclusion criteria and completed the required final clinical and radiographic follow-up. Final clinical and radiographic follow-up assessments were completed through May 2026. Due to the study’s retrospective nature and the anonymized evaluation of clinical and radiographic information, the ethics committee waived the need for obtaining written informed consent.

**Figure 1:**
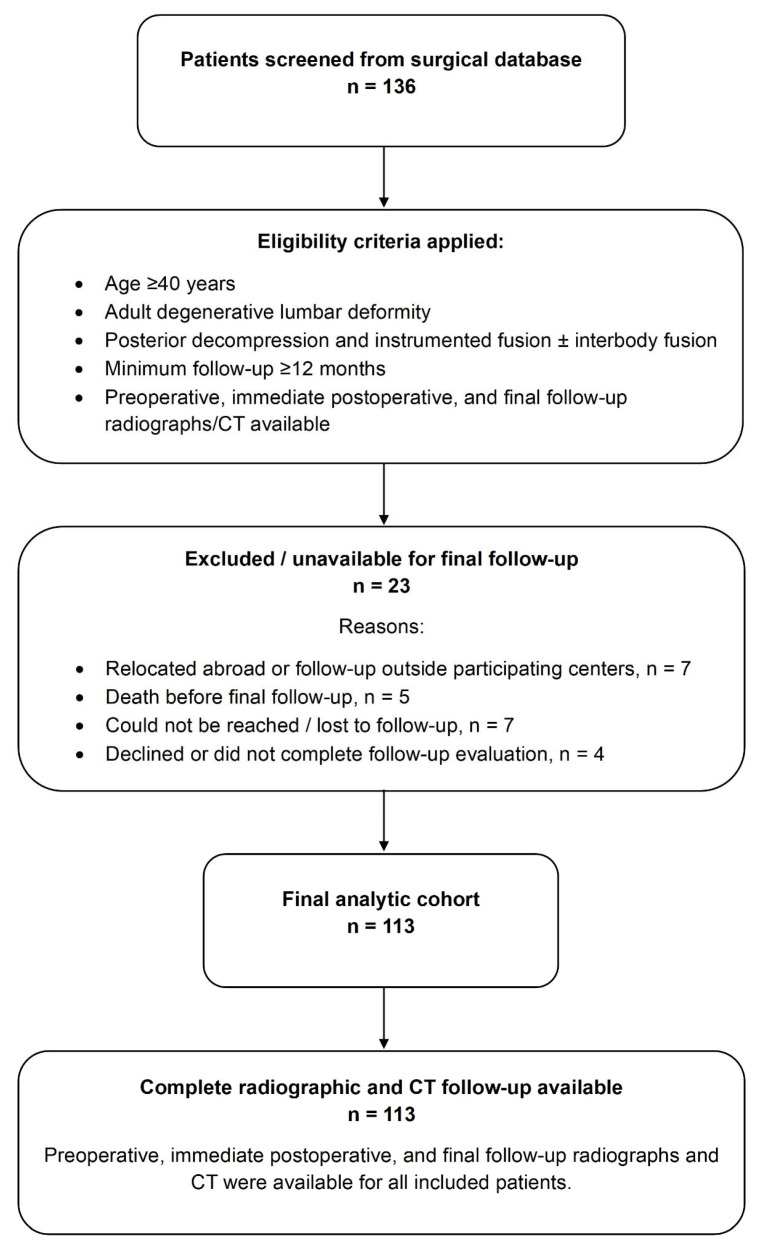
Patient selection flow diagram. Flow diagram showing the screened population, main eligibility criteria, reasons for exclusion or unavailability for final follow-up, and formation of the final analytic cohort. Of 136 screened patients, 23 were excluded or unavailable for final follow-up, and 113 patients completed final clinical and radiographic follow-up and were included in the analysis.

A total of 136 patients were identified from the surgical databases of the participating centers. Twenty-three patients were not included in the final analytic cohort because final follow-up could not be completed or was unavailable due to relocation abroad or follow-up outside the participating centers, death before follow-up, inability to be reached, loss to follow-up, or incomplete follow-up evaluation. The remaining 113 patients completed final follow-up and constituted the final analytic cohort. The patient selection process is shown in Figure 1.

A minimum follow-up period of 12 months was required. Final clinical follow-up was defined as the last available outpatient evaluation or structured follow-up contact. Final radiographic follow-up was defined as the last available postoperative radiographic and computed tomography (CT) assessment.

Follow-up adequacy was evaluated according to two predefined criteria: completion of both final clinical and final radiographic assessment in the analytic cohort and a minimum 12-month interval after surgery. Patients who did not meet these criteria were not included in the final analytic cohort, and follow-up duration was reported as mean ± standard deviation, median with interquartile range, and range.

The objective of this study was to evaluate radiographic spinopelvic realignment, clinical outcomes, postoperative complications, and predictors of unfavorable postoperative course in patients undergoing surgical correction for adult degenerative lumbar deformity. The study specifically examined whether baseline patient factors, fusion extent, and immediate postoperative residual PI–LL mismatch were associated with the final clinical course.

The study was conducted in accordance with the Declaration of Helsinki and was approved by the Non-Interventional Clinical Research Ethics Committee of Istanbul Aydın University (approval number: 178/2026; date: 6 May 2026).

Ethics committee approval was obtained after the surgical study period because the study was designed as a retrospective review of pre-existing clinical and radiographic data. The approved protocol covered anonymized retrospective evaluation of patients treated during the study period, and no prospective intervention or change in clinical management was performed for the purposes of this study.

### 2.2. Patient Population

Adult degenerative lumbar deformity was defined as a degenerative lumbar or thoracolumbar spinal condition associated with one or more deformity-related features, including degenerative lumbar scoliosis, lumbar spinal stenosis with deformity, degenerative spondylolisthesis, sagittal malalignment, coronal malalignment, PI–LL mismatch, or loss of lumbar lordosis. These categories were not mutually exclusive.

Surgery was considered in patients with disabling back or leg pain, neurogenic claudication, progressive neurological symptoms, functional limitation, or clinically relevant sagittal or coronal malalignment that persisted despite conservative treatment or required operative correction according to the treating surgeon’s judgment.

Patients were included if they were aged ≥40 years, had adult degenerative lumbar deformity, underwent posterior decompression and instrumented fusion with or without interbody fusion, and had available preoperative, immediate postoperative, and final follow-up radiographs and CT images. Patients were excluded if they had deformities secondary to tumors, active infections, acute trauma, neuromuscular diseases, congenital deformities, or ankylosing spondylitis. Patients treated with isolated decompression without instrumentation, those with incomplete imaging at any predefined time point, and those without completed final follow-up were also excluded.

Osteoporosis was defined as a preoperative dual-energy X-ray absorptiometry T-score ≤ −2.5 at the lumbar spine, femoral neck, or total hip, or as a previously documented diagnosis of osteoporosis with anti-osteoporotic medication use recorded in the medical history.

### 2.3. Surgical Treatment

The surgical strategy was determined by the treating surgeon at each center according to symptoms, neurological findings, deformity pattern, sagittal and coronal alignment, bone quality, comorbidities, and overall surgical risk. Surgery generally consisted of posterior decompression, multilevel pedicle screw instrumentation, deformity correction, and posterolateral fusion.

Although no uniform operative protocol was imposed across centers, surgical planning followed similar clinical and radiographic principles, including decompression, deformity correction, restoration of sagittal and coronal alignment, stabilization of degenerative instability, and solid fusion. Pelvic fixation was used at the discretion of the treating surgeon, particularly in cases requiring long fusion to the sacrum or pelvis, poor bone quality, marked sagittal imbalance, lumbosacral instability or degeneration, revision surgery, or enhanced distal construct stability.

Interbody fusion was performed when needed for anterior column support, restoration of disc height, foraminal decompression, segmental lordosis correction, or stabilization of degenerative instability. Transforaminal lumbar interbody fusion (TLIF) was the predominant interbody technique in this cohort.

Recorded operative variables included the number of fused and decompressed levels, interbody fusion levels, upper and lower instrumented vertebrae, distal fixation level, pelvic fixation, construct type, interbody fusion technique, osteotomy type, operative time, and estimated blood loss.

### 2.4. Radiographic Assessment

Radiographic assessment was performed at three predefined time points: preoperatively, immediately postoperatively, and at final follow-up. All patients in the final analytic cohort had radiographs and CT available at each time point. Full-length standing anteroposterior and lateral radiographs were available for all included patients at the predefined radiographic time points and were used for global coronal and sagittal alignment measurements.

Radiographic measurements were performed by a single reviewer experienced in spinal radiographic assessment using standardized definitions at the predefined time points. To assess intraobserver reliability, the main radiographic parameters were remeasured by the same reviewer in a randomly selected subset of 30 patients. Reliability was evaluated using the intraclass correlation coefficient.

Preoperative full-length standing anteroposterior and lateral radiographs were used to characterize baseline coronal and sagittal deformity. Immediate postoperative radiographs were used to assess early alignment, while immediate postoperative CT was used to evaluate screw placement, interbody cage position, construct integrity, and early implant-related findings. Final follow-up radiographs and CT were used to evaluate maintenance of correction, fusion-related status, implant integrity, and delayed mechanical complications.

The following radiographic parameters were recorded: coronal Cobb angle, lumbar lordosis, pelvic incidence, PT, sacral slope, PI–LL mismatch, SVA, TPA, coronal vertical axis, apical vertebral translation, and thoracic kyphosis. The PI–LL mismatch was calculated as pelvic incidence minus lumbar lordosis. Changes in radiographic parameters were calculated from preoperative to immediate postoperative assessment and from immediate postoperative assessment to final follow-up.

### 2.5. Clinical Follow-Up, PROM Assessment, and Outcome Definitions

Clinical outcome assessments included final clinical status, persistent or recurrent symptoms, functional deterioration, postoperative complications, and revision or reoperation. All patients in the final analytic cohort were contacted and invited for final outpatient clinical and radiological follow-up. Final clinical status was assessed during outpatient evaluation or structured follow-up contact, whereas final radiographic assessment was based on available full-length standing radiographs and CT.

To strengthen interpretation of functional and symptomatic outcomes, final clinical status was interpreted in parallel with PROM change. Clinical categories captured improvement, residual pain, functional limitation, unchanged status, and worsening, whereas PROMs quantified disability, back pain, leg pain, and deformity-specific quality of life when paired data were available.

Available patient-reported outcome measures (PROMs) included the Oswestry Disability Index (ODI), visual analog scale (VAS) scores for back and leg pain, and the Scoliosis Research Society-22 (SRS-22) total score. Minimum clinically important difference (MCID) achievement was recorded when available. PROM analyses were performed using an available case approach. Preoperative and final follow-up values were compared only among patients with paired data for the corresponding outcome, and the denominator was reported separately for each score. No last observation was carried forward or multiple imputation was performed.

Improvement was calculated as preoperative minus final follow-up value for ODI and VAS scores, and as final follow-up minus preoperative value for SRS-22 total score. Thus, positive values represented improvement for all PROM improvement variables.

An unfavorable postoperative course was defined as persistent or worsened pain with functional limitation, symptomatic mechanical complication, deep infection requiring surgical treatment, or revision/reoperation. Revision/reoperation was considered an unfavorable event for clinical outcome classification but was also analyzed separately as a surgical endpoint. It was not included within the composite definition of late/mechanical complications. Asymptomatic radiographic findings were not classified as unfavorable unless associated with symptoms, functional deterioration, instability, loss of correction, or surgical reintervention.

### 2.6. Complications and Revision/Reoperation Endpoint

Postoperative complications were classified as early complications and late/mechanical complications. Early complications included dural tear, postoperative cerebrospinal fluid leakage requiring reoperation, infection, urinary tract infection, pneumonia, thromboembolic events, cardiac complications, delirium, and early neurological deficit.

Late/mechanical complications included proximal junctional kyphosis (PJK), proximal junctional failure (PJF), pseudarthrosis, rod fracture, implant failure, screw loosening, vertebral compression fracture, and loss of correction. Revision/reoperation was analyzed as a separate surgical endpoint and included mechanical revision, infection-related debridement or revision, and reoperation related to dural tear or postoperative cerebrospinal fluid leakage.

Complications were identified and classified by the treating surgeons at each participating center based on operative reports, inpatient records, outpatient follow-up notes, imaging studies, and revision/reoperation records. For the present analysis, complication data were reviewed during the data extraction process to ensure consistency with the predefined complication categories.

### 2.7. Missing Data and Bias

Clinical and radiographic follow-up data were available for all 113 patients in the final analytic cohort. Therefore, radiographic analyses were performed in the full cohort. Missing data mainly involved incomplete documentation of patient-reported outcome measures. Available baseline demographic, clinical, and preoperative radiographic data of patients excluded or unavailable for final follow-up were reviewed to assess potential attrition bias. Final clinical, radiographic, and PROM outcomes could not be evaluated in this group because final follow-up data were unavailable.

For each PROM, analyses were restricted to patients with available paired preoperative and final follow-up data. To assess potential bias related to incomplete ODI documentation, patients with and without available final ODI data were compared in a supplementary analysis. A supplementary center-based comparison was also performed to assess baseline, operative, radiographic, and clinical comparability across the three participating centers.

Potential sources of bias included the retrospective design, multicenter data collection, heterogeneity in deformity patterns and surgical strategies, and incomplete PROM documentation in a subset of patients. These were addressed using predefined eligibility criteria, standardized radiographic and clinical variables, separate reporting of denominators for available-case analyses, and separate analysis of revision/reoperation as a surgical endpoint.

### 2.8. Statistical Analysis

Continuous variables are reported as mean ± standard deviation or median with interquartile range according to distribution. Categorical variables are reported as frequencies and percentages. Normality was assessed using the Shapiro–Wilk test and visual inspection of histograms and Q–Q plots.

Radiographic changes were analyzed using predefined paired comparisons between preoperative and immediate postoperative measurements and between immediate postoperative and final follow-up measurements. Paired-samples t tests or Wilcoxon signed-rank tests were used according to distributional assumptions. A Bonferroni-corrected significance threshold of *p* < 0.025 was applied for the two planned radiographic comparisons.

To assess changes in PROMs, paired-samples t tests or Wilcoxon signed-rank tests were employed for patients with available paired data, with *p* < 0.05 considered statistically significant. To compare subgroups, independent-samples t tests, Mann–Whitney U tests, chi-square tests, Fisher’s exact tests, or Fisher–Freeman–Halton exact tests were utilized as suitable. Center-based comparisons were performed using one-way ANOVA or Kruskal–Wallis tests for continuous variables and chi-square or exact tests for categorical variables.

Correlations between immediate radiographic correction and clinical improvement were evaluated using Pearson or Spearman correlation analysis according to distribution. Logistic regression analysis was performed to identify factors associated with unfavorable postoperative course. Univariable logistic regression was first performed for clinically relevant candidate predictors. The final multivariable model included age, osteoporosis, number of fusion levels, and immediate postoperative PI–LL mismatch. This restricted model was selected to reduce overfitting and to prioritize clinically relevant baseline, surgical, and early postoperative radiographic predictors. ORs for PI–LL mismatch were reported per 10° increase.

All tests were two-sided, and *p* < 0.05 was considered statistically significant unless otherwise specified. Statistical analyses were performed using Jamovi version 2.6.44. During the preparation of this manuscript, the authors used ChatGPT (OpenAI, GPT-5.5) for language editing, formatting assistance, and manuscript organization support.

## 3. Results

### 3.1. Patient Selection and Baseline Characteristics

A total of 136 consecutive patients who underwent surgery for adult degenerative lumbar deformity were screened. Twenty-three patients were excluded or were unavailable for the final follow-up, leaving 113 patients in the final analytic cohort. All included patients had completed clinical follow-up and had available preoperative, immediate postoperative, and final follow-up radiographic and CT assessments. The patient selection process is shown in Figure 1.

Within the final analytic cohort, follow-up was complete according to the predefined study requirements: 113 of 113 patients had final clinical assessment and final full-length standing radiographs and CT, with a minimum follow-up of 12 months. Follow-up du-ration was 31.0 ± 12.9 months, with a median of 31 months, an interquartile range of 19–42 months, and a range of 12–63 months. These data support the adequacy of mid-term outcome assessment for the predefined clinical, radiographic, complication, and revision/reoperation endpoints.

Baseline characteristics are summarized in Table 1. The mean age was 63.5 ± 7.8 years, and 76.1% (*n* = 86) of patients were female. The mean BMI was 26.6 ± 3.3 kg/m^2^. The cohort had a moderate comorbidity burden, with diabetes in 23.9% (*n* = 27), smoking in 31.9% (*n* = 36), osteoporosis in 31.9% (*n* = 36), and previous lumbar surgery in 17.7% (*n* = 20). The mean follow-up duration was 31.0 ± 12.9 months.

The study population reflected a broad spectrum of degenerative lumbar deformities. Lumbar stenosis with deformity, loss of lumbar lordosis, degenerative lumbar scoliosis, sagittal malalignment, coronal malalignment, and degenerative spondylolisthesis were all represented, and these categories were not mutually exclusive.

### 3.2. Operative Characteristics

Operative details are presented in Table 2. The mean number of fused levels was 7.5 ± 2.3. The upper instrumented vertebra was most commonly located in the lower thoracic or thoracolumbar region, whereas the lower instrumented vertebra was most frequently S1. Pelvic fixation was used in 17.7% (*n* = 20).

Most procedures were performed using a standard two-rod construct, whereas 23.0% (*n* = 26) received a four-rod or accessory rod construct. TLIF was the predominant interbody technique and was used in 91.2% (*n* = 103). Osteotomy was performed in 63.7% (*n* = 72), most commonly as Ponte osteotomy. The mean operative time was 360.3 ± 51.7 min, and the mean estimated blood loss was 998.6 ± 278.9 mL.

### 3.3. Radiographic Correction and Maintenance of Alignment

**Figure 2:**
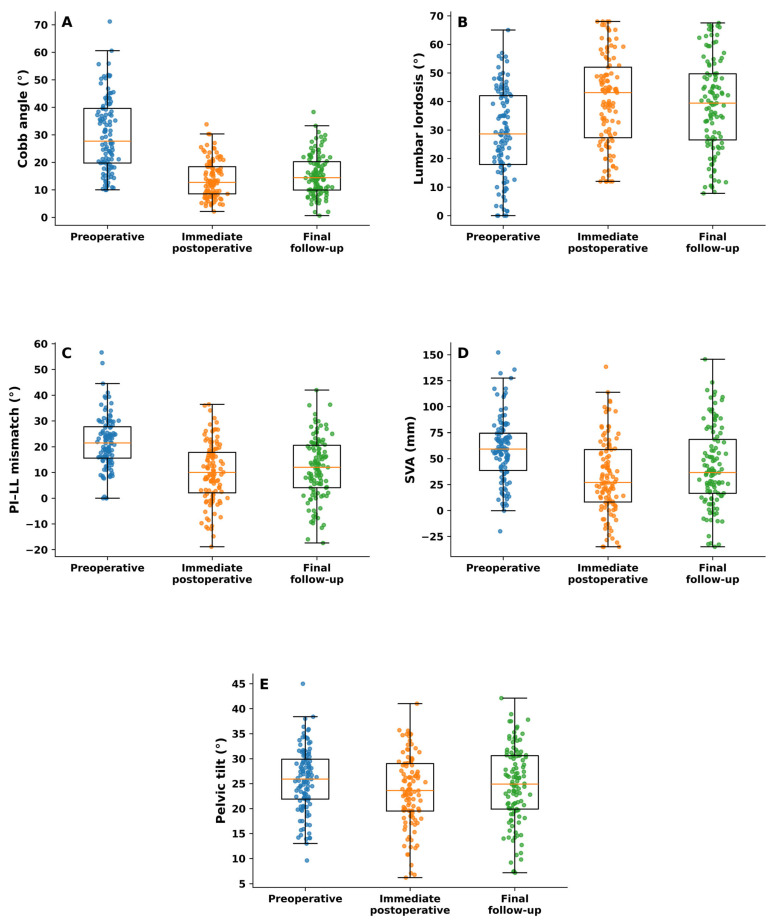
Radiographic changes from preoperative assessment to immediate postoperative assessment and final follow-up. Line plots showing changes in key coronal and sagittal radiographic parameters at the three predefined time points. (**A**) Coronal Cobb angle; (**B**) lumbar lordosis; (**C**) pelvic incidence–lumbar lordosis mismatch; (**D**) sagittal vertical axis; (**E**) pelvic tilt. Values are presented as mean values across the study cohort. PI–LL, pelvic incidence–lumbar lordosis mismatch; SVA, sagittal vertical axis.

Radiographic changes are summarized in Table 3 and illustrated in Figure 2. Surgery resulted in significant improvement in both coronal and sagittal alignment parameters from the preoperative assessment to the immediate postoperative evaluation.

The mean Cobb angle improved from 29.8 ± 13.1° to 13.7 ± 6.7° immediately after surgery (Figure 2A). Lumbar lordosis increased from 28.8 ± 15.5° to 40.3 ± 16.0° (Figure 2B), while PI–LL mismatch decreased from 21.7 ± 10.0° to 10.1 ± 11.5° (Figure 2C). SVA also improved, decreasing from 58.8 ± 31.4 mm to 32.5 ± 36.0 mm (Figure 2D). PT showed a smaller but statistically significant reduction (Figure 2E).

At the final follow-up, partial loss of correction was observed across several parameters, although alignment generally remained improved compared with baseline. Cobb angle, PI–LL mismatch, SVA, and PT increased slightly from the immediate postoperative values, while lumbar lordosis decreased modestly. These final follow-up changes were statistically significant but relatively small in magnitude.

Intraobserver reliability for the main radiographic parameters was excellent, with intraclass correlation coefficients ranging from 0.919 to 0.969.

### 3.4. Clinical Outcomes and Patient-Reported Outcome Measures

Clinical outcomes and PROMs are shown in Table 4. At final follow-up, clinical status was classified as improved in 29.2% (*n* = 33), improved with mild residual symptoms in 11.5% (*n* = 13), improved despite persistent symptoms in 25.7% (*n* = 29), unchanged in 16.8% (*n* = 19), and worsened in 16.8% (*n* = 19). Overall, 66.4% (*n* = 75) showed some degree of clinical improvement. Persistent or recurrent symptoms with functional limitation were recorded separately and were present in 35.4% (*n* = 40). Overall, 35.4% (*n* = 40) were classified as having an unfavorable postoperative course. The symptomatic and functional component of this composite endpoint is shown in Table 4, while postoperative complications and revision/reoperation endpoints are detailed separately in Table 5.

PROM analyses were performed using available paired data for each outcome. ODI improved from 57.8 ± 12.6 preoperatively to 34.7 ± 8.7 at final follow-up, with a mean improvement of 23.1 ± 13.2 points. ODI MCID was achieved in 84.2% (*n* = 80) of patients with available ODI data. VAS back pain, VAS leg pain, and SRS-22 total scores also improved significantly, with MCID achievement rates of 72.4%, 89.8%, and 94.6%, respectively.

In addition to the mean PROM changes, the clinical categories provide a symptomatic interpretation of recovery. Forty-six patients (40.7%) were improved or improved with only mild residual symptoms, whereas 29 patients (25.7%) improved despite persistent symptoms. Thus, symptomatic benefit was not binary; a substantial proportion of patients experienced meaningful improvement but continued to have residual symptoms. This distinction is important because MCID achievement and unfavorable postoperative course were not mutually exclusive.

### 3.5. Complications and Revision/Reoperation

Postoperative complications and revision/reoperation outcomes are summarized in Table 5. Any postoperative complication occurred in 42.5% (*n* = 48). Early complications occurred in 27.4% (*n* = 31), while late or mechanical complications occurred in 29.2% (*n* = 33).

The most common early complications were dural tear, deep infection, superficial infection, urinary tract infection, delirium, pneumonia, and neurologic deficit. Late or mechanical complications most frequently included PJK, screw loosening, pseudarthrosis, vertebral compression fracture, implant failure, and rod fracture.

Revision or reoperation was analyzed as a separate surgical endpoint and occurred in 23.9% (*n* = 27). According to the primary indication, revision/reoperation was performed for mechanical causes in 15.9% (*n* = 18), infection-related causes in 6.2% (*n* = 7), and dural tear or CSF leak-related causes in 1.8% (*n* = 2).

The complication profile therefore reflected both early perioperative morbidity and delayed mechanical failure. The relatively high revision/reoperation rate should be interpreted together with the long constructs, frequent osteotomy use, and older degenerative population, rather than as isolated adverse events. These complications were clinically relevant because they contributed to the composite unfavorable postoperative course when associated with symptoms, functional deterioration, infection requiring surgery, or revision/reoperation.

### 3.6. Correlation Between Radiographic Correction and Clinical Improvement

Supplementary correlation analyses are shown in Supplementary Table S1. Overall, correlations between immediate radiographic correction and ODI improvement were weak. Cobb angle reduction showed a weak positive association with ODI improvement but did not reach statistical significance.

Some radiographic correction parameters were more closely related to pain improvement. In particular, SVA reduction showed a moderate correlation with VAS back pain improvement, while lumbar lordosis increase and PI–LL reduction showed weak but statistically significant correlations with VAS back improvement. No consistent meaningful association was observed between radiographic correction and VAS leg pain or SRS-22 improvement.

### 3.7. Predictors of Unfavorable Postoperative Course

Logistic regression analyses are presented in Table 6. In univariable analysis, osteoporosis, number of fused levels, preoperative PI–LL mismatch, and immediate postoperative PI–LL mismatch were associated with unfavorable postoperative course.

**Figure 3:**
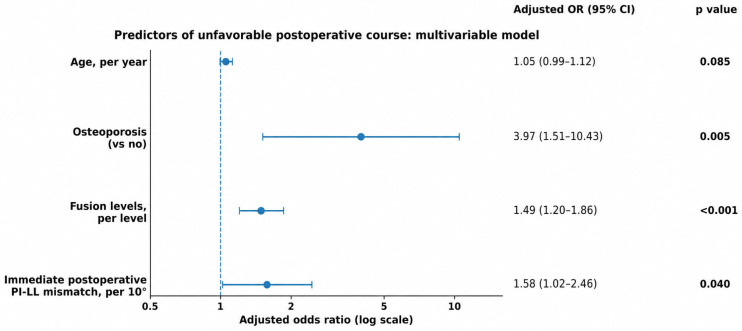
Multivariable predictors of unfavorable postoperative course. Forest plot showing adjusted odds ratios and 95% confidence intervals from the multivariable logistic regression model for unfavorable postoperative course. The dashed vertical line indicates the reference value of OR = 1. The model included age, osteoporosis, number of fused levels, and immediate postoperative PI–LL mismatch. Odds ratios for PI–LL mismatch are reported per 10° increase. CI, confidence interval; OR, odds ratio; PI–LL, pelvic incidence–lumbar lordosis mismatch.

In the multivariable model, osteoporosis remained independently associated with unfavorable postoperative course (adjusted odds ratio [OR], 3.97; 95% confidence interval [CI], 1.51–10.43; *p* = 0.005). The number of fused levels was also independently associated with an unfavorable postoperative course (adjusted OR per level, 1.49; 95% CI, 1.20–1.86; *p* < 0.001). Immediate postoperative PI–LL mismatch remained an independent radiographic predictor when analyzed per 10° increase (adjusted OR, 1.58; 95% CI, 1.02–2.46; *p* = 0.040). Age showed a non-significant trend toward increased odds of unfavorable postoperative course. The adjusted ORs from the multivariable model are shown in Figure 3.

### 3.8. Supplementary Analyses

Patients with and without available final ODI data are compared in Supplementary Table S2. Baseline demographic, operative, radiographic, and complication-related variables were broadly comparable between groups, supporting the available-case approach for PROM analyses.

Center-based comparisons are shown in Supplementary Table S3. Baseline demographic, operative, radiographic, and outcome variables were generally comparable across the three participating centers. Preoperative ODI differed modestly between centers, but no major center-level imbalance was observed in the main surgical, radiographic, complication, revision/reoperation, or unfavorable outcome variables.

## 4. Discussion

### 4.1. Principal Findings

In this three-center retrospective cohort, surgery for adult degenerative lumbar deformity was associated with significant improvement in coronal and sagittal radiographic alignment, along with clinically meaningful improvement in patients with available paired PROM data. However, the postoperative course was not uniformly favorable. Complications and revision/reoperation remained clinically relevant, reflecting the complexity of long-segment deformity correction in an older degenerative population. Therefore, the results should be interpreted as demonstrating both the potential benefit and the substantial morbidity of surgical reconstruction in this population.

The main finding of this study was that osteoporosis, greater number of fused levels, and higher immediate postoperative PI–LL mismatch were independently associated with an unfavorable postoperative course. Importantly, the regression model focused on baseline patient factors, operative burden, and early postoperative alignment, rather than using later outcome events as predictors. These findings suggest that outcome after adult degenerative lumbar deformity surgery is shaped not only by whether correction is achieved, but by the combined effect of residual postoperative alignment, bone quality, and the extent of surgical reconstruction.

The present study adds to the existing literature in several ways. First, it focuses on immediate postoperative PI–LL mismatch as an early marker of residual sagittal malalignment, rather than limiting the analysis to baseline deformity or final radiographic status. Second, it evaluates this early alignment marker together with osteoporosis and fusion length, integrating patient-related risk, surgical burden, and postoperative radiographic correction within the same clinical framework. Third, it separates patient-reported improvement from the overall postoperative course by considering persistent symptoms, complications, and revision/reoperation as related but distinct outcomes. This distinction is important because patients undergoing surgery for adult degenerative lumbar deformity may experience meaningful symptomatic improvement while still facing a considerable risk of mechanical complications and additional surgery.

### 4.2. Spinopelvic Realignment and Clinical Outcomes

The significance of sagittal alignment in adult spinal deformity is well recognized. Positive sagittal balance has been associated with worse HRQOL, and spinopelvic parameters such as SVA, PT, and PI–LL mismatch have become central radiographic markers in adult deformity assessment [11,12]. The SRS–Schwab classification further incorporated these sagittal modifiers into a clinically meaningful framework, showing that worse modifier grades correlate with poorer HRQOL and greater likelihood of operative treatment [13].

Our findings are consistent with this hypothesis. Surgery improved the Cobb angle, lumbar lordosis, PI–LL mismatch, SVA, and PT, and these radiographic changes were accompanied by significant improvement in ODI, VAS back pain, VAS leg pain, and SRS-22 scores. Similar improvements after degenerative lumbar deformity surgery have been reported in studies evaluating multisegment TLIF with Ponte osteotomy, long-level fusion strategies, and OLIF-based correction in degenerative lumbar scoliosis or adult degenerative lumbar deformity [2,17,23].

Functional and symptomatic outcomes in this cohort were therefore best understood as multidimensional. ODI, VAS back, VAS leg, and SRS-22 all improved significantly among patients with paired data, indicating measurable functional and pain-related benefit. However, final clinical status showed that recovery often occurred with residual symptoms, and 35.4% of patients had persistent or recurrent symptoms with functional limitation. This pattern suggests that the clinical value of surgery should not be judged solely by radiographic correction or average PROM improvement; the durability of symptom relief and the burden of residual functional limitation are equally important.

At the same time, the correlation between radiographic correction and clinical improvement in our cohort was not uniformly strong. This finding should be interpreted in light of the heterogeneity of adult degenerative lumbar deformity. The present cohort included patients with degenerative lumbar scoliosis, lumbar stenosis with deformity, degenerative spondylolisthesis, sagittal malalignment, coronal malalignment, and loss of lumbar lordosis, and these conditions frequently overlapped. Therefore, clinical improvement may reflect not only spinopelvic realignment, but also neural decompression, stabilization of degenerative instability, pain-generator modification, baseline disability, and the patient’s compensatory capacity. This heterogeneity may partly explain why correlations between radiographic correction and PROM improvement were modest. This interpretation is consistent with previous studies showing that the relationship between sagittal alignment and HRQOL is multifactorial. Takemoto et al. [14] showed that preoperative HRQOL is influenced by multiple demographic and clinical factors and is not always strongly explained by sagittal parameters alone. Similarly, Gao et al. [1] reported only weak correlations between spinopelvic parameters and pretreatment HRQOL in de novo degenerative lumbar scoliosis with lumbar stenosis, although LL, SVA, T1PA, and PI–LL remained clinically relevant. More recently, Zhang et al. [3] found that postoperative sagittal parameters, especially PI–LL, SVA, TPA, and global tilt, were more closely related to HRQOL after long-level fusion in DLS.

Nevertheless, one clinically relevant exception was observed in our cohort: preoperative-to-immediate postoperative SVA reduction demonstrated a moderate correlation with baseline-to-final VAS back pain improvement (ρ = 0.54, *p* < 0.001). This finding reinforces the importance of achieving adequate global sagittal realignment, particularly for mechanical back pain relief, even when correlations with broader functional outcomes such as ODI are more modest.

The clinical relevance of spinopelvic correction should also be considered beyond static radiographic parameters, because spinopelvic imbalance may influence posture, gait mechanics, functional recovery, and quality of life. Although not specific to adult degenerative lumbar deformity, translational scoliosis models and orthopedic studies on spinopelvic balance, gait, and quality of life support the broader concept that spinal and pelvic alignment has functional consequences across musculoskeletal conditions [24,25].

Taken together, these findings support a balanced interpretation. Radiographic correction is important, but postoperative recovery is influenced by more than alignment alone. Neural decompression, baseline disability, pain phenotype, complications, frailty, bone quality, and patient expectations all likely contribute to the final clinical course.

### 4.3. Immediate Postoperative PI–LL Mismatch as a Risk Marker

One of the most clinically relevant findings of this study was the independent association between immediate postoperative PI–LL mismatch and unfavorable postoperative course. Each 10° increase in residual immediate postoperative PI–LL mismatch increased the odds of an unfavorable course, even after adjustment for age, osteoporosis, and fusion length.

This finding supports the concept that early residual sagittal malalignment may have prognostic value. Liu et al. [15] specifically focused on immediate postoperative changes in spinopelvic parameters and found that ΔSVA predicted mid-term improvement in ODI and VAS scores among patients with adult degenerative scoliosis. Our results extend this idea by showing that residual immediate postoperative PI–LL mismatch is associated with a broader unfavorable postoperative course, not only with PROM change.

The importance of lordosis restoration is also supported by studies focusing on lower lumbar and global spinopelvic realignment. Zhang et al. [16] demonstrated that correction of lower lumbar lordosis was associated with changes in LL, PI–LL, SVA, and TPA after DLS surgery. Zhang et al. [17] also showed that LL correction strongly correlated with changes in SS, PT, SVA, TPA, and PI–LL after long sacroiliac fusion in DLS. These data reinforce the surgical relevance of achieving adequate postoperative lordosis relative to pelvic incidence.

Therefore, immediate postoperative PI–LL mismatch may be a practical early marker of residual alignment risk. Because it can be assessed soon after surgery, it may help identify patients who require closer surveillance, focused rehabilitation, bone health optimization, or earlier evaluation if symptoms recur.

Residual PI–LL mismatch may indicate inadequate restoration of lumbar lordosis relative to pelvic morphology. This residual malalignment can maintain compensatory pelvic retroversion and increased paraspinal muscle demand, while also increasing mechanical stress across the lumbosacral junction, implants, and adjacent segments. In patients with poor bone quality or limited compensatory reserve, this may contribute to persistent mechanical pain, loss of correction, implant-related complications, or revision surgery. Therefore, immediate postoperative PI–LL mismatch may serve as an early marker of persistent biomechanical overload rather than a purely radiographic finding.

### 4.4. Osteoporosis, Fusion Length, and Mechanical Complications

Osteoporosis was the strongest patient-related predictor of unfavorable postoperative course in our multivariable model. This finding is clinically relevant. Poor bone quality can compromise screw fixation, increase the risk of vertebral compression fracture, contribute to proximal junctional problems, and affect construct durability. Prior meta-analyses and reviews have similarly identified osteoporosis or osteopenia as important risk factors for PJK, PJF, and mechanical complications after adult deformity surgery [8,9,26].

Osteoporosis may worsen the postoperative course through several mechanical pathways. Poor bone quality can reduce screw purchase, increase the risk of screw loosening and vertebral compression fracture, impair fusion, and contribute to loss of correction or implant-related failure. These risks are particularly relevant in long constructs, where greater lever arms increase stress at the lumbosacral and junctional regions. Therefore, osteoporosis should be viewed as a modifiable risk factor that may affect both construct durability and clinical recovery.

Fusion length was also independently associated with unfavorable postoperative course. Longer constructs are often necessary in patients with more extensive deformity, but they also increase surgical invasiveness, junctional stress, and mechanical demand across the construct. Lamas et al. [7] showed that degenerative scoliosis correction extending to the pelvis carries substantial risk for non-union, rod-related failure, and revision, particularly with traditional two-rod constructs. Raganato et al. [10] further emphasized that mechanical complications after adult deformity surgery should not be considered a single homogeneous entity, because proximal junctional problems and fusion failure show different time-to-onset patterns and risk profiles.

These findings are consistent with our results. The number of fused levels should not only be viewed as a technical variable or marker of deformity severity. It also reflects the mechanical and biological burden placed on the patient after reconstruction.

The complication data add clinically important context to these predictors. Early complications, including dural tear, infection, pulmonary or urinary complications, delirium, and neurologic deficit, reflect the perioperative vulnerability of this population and the invasiveness of long-segment reconstruction. In contrast, late/mechanical complications such as PJK, pseudarthrosis, screw loosening, vertebral compression fracture, implant failure, and rod fracture reflect the difficulty of maintaining correction over time. This distinction is clinically relevant because prevention strategies differ. Early morbidity may be reduced through perioperative optimization, careful patient selection, and meticulous surgical technique, whereas late mechanical events require attention to bone quality, construct design, alignment targets, junctional protection, and long-term surveillance [7,8,9,10,26].

### 4.5. Clinical Implications

The practical message of this study is not that all patients should undergo more aggressive correction. However, correction should be individualized. Residual PI–LL mismatch may increase the risk of persistent symptoms and unfavorable postoperative course, but excessive correction may also increase mechanical risk, especially in older or osteoporotic patients.

This balance has become increasingly important in the age-adjusted alignment literature. Lafage et al. [18] emphasized that ideal alignment targets should vary with age, and Byun et al. [20] showed that overcorrection relative to age-adjusted PI–LL goals was associated with a higher incidence of PJK. Therefore, surgical planning should aim for sufficient spinopelvic realignment while considering age, pelvic morphology, flexibility, bone quality, construct length, and compensatory capacity.

From a clinical standpoint, our findings support three priorities: careful preoperative bone health assessment, risk-aware planning of fusion length and distal fixation, and early postoperative evaluation of residual PI–LL mismatch. In patients with osteoporosis or high mechanical risk, optimization of bone health before surgery should be considered whenever clinically feasible, including DXA-based assessment, correction of vitamin D or calcium deficiency, anti-osteoporotic treatment, and consideration of anabolic therapy in selected high-risk patients according to current bone health recommendations. Patients with osteoporosis, long constructs, or residual mismatch may benefit from closer follow-up and earlier attention to mechanical symptoms.

### 4.6. Limitations

This study has several limitations. First, its retrospective design limits causal inference and introduces the possibility of selection and documentation bias. Second, although the study was conducted across three centers, surgical techniques, implant selection, and postoperative management were not fully standardized. This reflects real-world practice but also introduces heterogeneity. Although intraobserver reliability was assessed and found to be excellent, radiographic measurements were performed by a single reviewer, and interobserver reliability was not formally evaluated.

With respect to follow-up adequacy, all included patients fulfilled the predefined minimum 12-month follow-up requirement and completed final clinical and radio-graphic/CT assessment; thus, follow-up was complete for the final analytic cohort and adequate for the mid-term endpoints analyzed. Nevertheless, the mean follow-up of 31 months does not exclude very late mechanical complications, adjacent-segment degeneration, or late functional decline, and longer surveillance remains necessary.

Third, PROM data were incomplete in a subset of patients and were therefore analyzed using an available case approach. Although supplementary analyses were performed to assess potential differences between patients with and without available final ODI data, residual documentation bias cannot be excluded. Although available baseline information from excluded or unavailable patients was reviewed during screening, these patients lacked completed final follow-up assessments; therefore, the potential effect of attrition on final clinical and radiographic outcomes cannot be fully excluded. Fourth, final clinical follow-up included outpatient assessment and structured follow-up contact, whereas radiographic follow-up was based on available radiographs and CT.

Finally, the multivariable model was intentionally restricted to reduce overfitting. As a result, some potentially relevant factors, including frailty measures, detailed bone mineral density values, specific construct characteristics, UIV selection, rod material, and formal age-adjusted alignment scores, could not be fully investigated. Additionally, final outcome events such as revision/reoperation, late mechanical complications, and deep infection were not entered as predictors in the regression models, because these variables were part of, or closely linked to, the composite outcome definition. This approach was used to reduce circularity and structural overfitting, and to focus the analysis on baseline patient factors, operative burden, and early postoperative alignment.

### 4.7. Future Perspectives

Future prospective multicenter studies are needed to validate the prognostic role of immediate postoperative PI–LL mismatch in adult degenerative lumbar deformity surgery. Such studies should include standardized PROM collection, longer radiographic follow-up, formal assessment of age-adjusted alignment targets, and quantitative evaluation of bone quality. Further research should also examine how residual postoperative alignment interacts with frailty, osteoporosis treatment, construct characteristics, pelvic fixation, rod configuration, and lower-lumbar lordosis restoration. These data may help develop individualized risk-stratification models and support more patient-specific surgical planning in adult degenerative lumbar deformity.

## 5. Conclusions

In this three-center retrospective cohort of patients who underwent surgery for adult degenerative lumbar deformity, operative treatment was associated with significant radiographic correction and meaningful clinical improvement in patients with available paired PROM data. However, postoperative complications and revision/reoperation remained clinically important. Osteoporosis, greater fusion length, and residual immediate postoperative PI–LL mismatch were independently associated with an unfavorable postoperative course. The principal contribution of this study is that residual immediate postoperative PI–LL mismatch, when interpreted together with osteoporosis and fusion length, may serve as an early multidomain risk signal in a mid-term analytic cohort with complete clinical and radiographic follow-up. These findings support the importance of individualized spinopelvic realignment, bone health optimization, and risk-aware surgical planning for adult degenerative lumbar deformity surgery.

## Figures and Tables

**Table 1:** Baseline demographic, clinical, and degenerative deformity characteristics of the final analytic cohort.

Variable	Total Cohort (*n* = 113)
**Study center**	
Center 1	36 (31.9)
Center 2	38 (33.6)
Center 3	39 (34.5)
**Demographic characteristics**	
Age, years	63.5 ± 7.8
Female sex	86 (76.1)
Male sex	27 (23.9)
BMI, kg/m^2^	26.6 ± 3.3
**Clinical status and comorbidities**	
ASA I	5 (4.4)
ASA II	76 (67.3)
ASA III	30 (26.5)
ASA IV	2 (1.8)
Diabetes mellitus	27 (23.9)
Smoking	36 (31.9)
Osteoporosis	36 (31.9)
Previous lumbar surgery	20 (17.7)
**Follow-up**	
Follow-up duration, months, mean ± SD; range	31.0 ± 12.9; range, 12–63
Follow-up duration, months, median [IQR]	31 [19–42]
Completed clinical and radiographic follow-up	113 (100.0)
**Degenerative phenotype and deformity features**	
Degenerative lumbar scoliosis	68 (60.2)
Lumbar stenosis with deformity	82 (72.6)
Degenerative spondylolisthesis	47 (41.6)
Sagittal malalignment	64 (56.6)
Coronal malalignment	58 (51.3)
Loss of lumbar lordosis	80 (70.8)
Preoperative PI–LL mismatch >10°	100 (88.5)

Notes: Data are presented as mean ± standard deviation, median [interquartile range], or *n* (%) unless otherwise indicated. Diagnostic and radiographic phenotype categories are not mutually exclusive because patients could present with more than one degenerative deformity feature. ASA, American Society of Anesthesiologists; BMI, body mass index; PI–LL, pelvic incidence–lumbar lordosis.

**Table 2:** Operative and perioperative characteristics of the final analytic cohort.

Variable	Total Cohort (*n* = 113)
**Operative setting**	
Center 1	36 (31.9)
Center 2	38 (33.6)
Center 3	39 (34.5)
**Surgical construct and fusion**	
Number of fused levels	7.5 ± 2.3; range, 2–12
**UIV region**	
Upper thoracic	5 (4.4)
Lower thoracic	45 (39.8)
Thoracolumbar	40 (35.4)
Lumbar	23 (20.4)
**LIV**	
L5	17 (15.0)
S1	84 (74.3)
S2/ilium/pelvis	12 (10.6)
Pelvic fixation	20 (17.7)
**Construct type**	
2-rod construct	87 (77.0)
4-rod/accessory rod construct	26 (23.0)
**Interbody fusion and decompression**	
**Interbody fusion technique**	
TLIF	103 (91.2)
PLIF	3 (2.7)
OLIF	2 (1.8)
Mixed interbody techniques	5 (4.4)
Number of interbody levels	2.4 ± 0.8; range, 1–4
Number of decompressed levels	1.8 ± 0.9; range, 0–3
**Osteotomy type**	
None	41 (36.3)
Ponte osteotomy	58 (51.3)
Smith–Petersen osteotomy	11 (9.7)
Other posterior column osteotomy	3 (2.7)
Number of osteotomy levels	1.6 ± 1.4; range, 0–5
**Perioperative data**	
Operative time, min	360.3 ± 51.7; range, 229–486
Estimated blood loss, mL	998.6 ± 278.9; range, 367–1745
**Imaging availability**	
Preoperative standing radiographs	113 (100.0)
Preoperative computed tomography	113 (100.0)
Immediate postoperative radiographs	113 (100.0)
Immediate postoperative computed tomography	113 (100.0)
Final follow-up radiographs	113 (100.0)
Final follow-up computed tomography	113 (100.0)

Notes: Values are presented as mean ± standard deviation (range) or *n* (%). UIV, upper instrumented vertebra; LIV, lower instrumented vertebra; TLIF, transforaminal lumbar interbody fusion; PLIF, posterior lumbar interbody fusion; OLIF, oblique lumbar interbody fusion.

**Table 3:** Radiographic spinopelvic parameters before surgery, immediately after surgery, and at final follow-up.

Parameter	Preoperative (*n* = 113)	Immediate Postoperative (*n* = 113)	Final Follow-Up (*n* = 113)	Change Immediate–Preoperative	*p* Value	Change Final–Immediate	*p* Value
Coronal Cobb angle, °	29.8 ± 13.1	13.7 ± 6.7	15.4 ± 7.3	−16.1 ± 7.9	<0.001	1.7 ± 2.8	<0.001
CVA, mm	19.7 ± 12.4	17.0 ± 14.3	18.1 ± 14.7	−2.7 ± 7.7	<0.001	1.1 ± 4.5	0.009
AVT, mm	20.6 ± 12.9	14.6 ± 12.2	16.2 ± 12.1	−6.0 ± 5.5	<0.001	1.6 ± 3.1	<0.001
Thoracic kyphosis, °	28.2 ± 12.0	31.0 ± 14.2	32.5 ± 14.6	2.7 ± 6.8	<0.001	1.5 ± 4.4	<0.001
LL, °	28.8 ± 15.5	40.3 ± 16.0	38.7 ± 16.2	11.5 ± 7.0	<0.001	−1.6 ± 2.4	<0.001
PT, °	25.7 ± 6.3	23.6 ± 7.0	24.6 ± 7.6	−2.1 ± 2.7	<0.001	1.0 ± 2.0	<0.001
SS, °	24.7 ± 9.7	26.8 ± 10.0	25.8 ± 10.2	2.1 ± 2.7	<0.001	−1.0 ± 2.0	<0.001
PI–LL mismatch, °	21.7 ± 10.0	10.1 ± 11.5	11.6 ± 11.9	−11.7 ± 7.2	<0.001	1.6 ± 2.4	<0.001
SVA, mm	58.8 ± 31.4	32.5 ± 36.0	42.1 ± 38.2	−26.3 ± 22.4	<0.001	9.6 ± 13.0	<0.001
TPA, °	21.3 ± 4.5	19.7 ± 4.1	20.6 ± 4.6	−1.6 ± 4.3	<0.001	0.8 ± 3.1	0.005

Notes: Values are presented as mean ± standard deviation unless otherwise indicated. Change values were calculated as the later time point minus the earlier time point; therefore, negative values indicated a reduction in the Cobb angle, PI–LL mismatch, SVA, TPA, CVA, and AVT, whereas positive values indicate increase. *p* values were calculated using predefined paired comparisons with paired-samples t tests or Wilcoxon signed-rank tests according to distribution. A Bonferroni-corrected significance threshold of *p* < 0.025 was applied to the two planned pairwise radiographic comparisons. AVT, apical vertebral translation; CVA, coronal vertical axis; LL, lumbar lordosis; PI, pelvic incidence; PI–LL, pelvic incidence–lumbar lordosis mismatch; PT, pelvic tilt; SS, sacral slope; SVA, sagittal vertical axis; TPA, T1 pelvic angle.

**Table 4:** Clinical postoperative course and patient-reported outcome measures.

**A.** Postoperative course, total cohort (***n*** = 113)							
**Variable**					***n*** **(%)**		
Final clinical and radiographic follow-up completed					113 (100.0)		
**Final clinical status**							
Improved					33 (29.2)		
Improved with mild residual symptoms					13 (11.5)		
Improved but persistent symptoms					29 (25.7)		
Unchanged					19 (16.8)		
Worsened					19 (16.8)		
Persistent/recurrent symptoms with functional limitation					40 (35.4)		
Unfavorable postoperative course					40 (35.4)		
**B.** Patient-reported outcome measures, available case analysis							
**Outcome**	**Analyzed, *n***	**Preoperative**	**Final Follow-Up**	**Mean Improvement**		***p*** **Value**	**MCID Achieved, *n* (%)**
**ODI**	95	57.8 ± 12.6	34.7 ± 8.7	23.1 ± 13.2		<0.001	80 (84.2)
**VAS back**	98	6.7 ± 1.5	4.0 ± 1.8	2.8 ± 1.1		<0.001	71 (72.4)
**VAS leg**	98	6.0 ± 2.0	2.7 ± 1.9	3.2 ± 0.9		<0.001	88 (89.8)
**SRS-22 total**	93	2.1 ± 0.3	3.1 ± 0.5	1.0 ± 0.3		<0.001	88 (94.6)

Notes: Values are presented as *n* (%) or mean ± standard deviation unless otherwise indicated. Patient-reported outcome measures were analyzed using available-case analysis; *n* analyzed is reported separately for each outcome. Mean improvement was calculated as preoperative minus final follow-up value for ODI and VAS scores, and final follow-up minus preoperative value for SRS-22 total score. Unfavorable postoperative course and MCID achievement were not mutually exclusive; patients with complications or reoperation could still achieve clinically meaningful improvement in PROMs compared with baseline. *p* values are from paired-samples t tests or Wilcoxon signed-rank tests according to distributional assumptions; a two-sided *p* value < 0.05 was considered statistically significant. MCID, minimal clinically important difference; ODI, Oswestry Disability Index; PROM, patient-reported outcome measure; SRS-22, Scoliosis Research Society-22; VAS, visual analog scale.

**Table 5:** Postoperative complications and revision/reoperation endpoint.

Variable	Total Cohort (*n* = 113), *n* (%)
**A. Overall complications**	
Any postoperative complication	48 (42.5)
Any early complication	31 (27.4)
Any late/mechanical complication	33 (29.2)
**B. Early complications**	
Dural tear	8 (7.1)
Postoperative CSF leak requiring reoperation	2 (1.8)
Deep infection	7 (6.2)
Superficial infection	6 (5.3)
UTI	6 (5.3)
Pneumonia	4 (3.5)
DVT/PE	2 (1.8)
Cardiac complication	2 (1.8)
Delirium	5 (4.4)
Neurologic deficit	4 (3.5)
**C. Late/mechanical complications**	
Proximal junctional kyphosis	16 (14.2)
Proximal junctional failure	2 (1.8)
Pseudarthrosis	8 (7.1)
Rod fracture	5 (4.4)
Implant failure	6 (5.3)
Screw loosening	10 (8.8)
Vertebral compression fracture	7 (6.2)
**D. Revision/reoperation endpoint**	
Revision/reoperation endpoint, total	27 (23.9)
Mechanical revision/reoperation, primary indication	18 (15.9)
Infection-related debridement/revision, primary indication	7 (6.2)
Dural tear/CSF leak-related reoperation, primary indication	2 (1.8)

Notes: Values are presented as *n* (%). Early and late/mechanical complication categories were not mutually exclusive. Revision/reoperation was analyzed as a separate surgical endpoint and was not included in the definition of late/mechanical complications. Revision/reoperation primary indication categories were mutually exclusive. PJF is considered a severe subset/progression of PJK. CSF, cerebrospinal fluid; DVT/PE, deep vein thrombosis/pulmonary embolism; PJK, proximal junctional kyphosis; PJF, proximal junctional failure; UTI, urinary tract infection.

**Table 6:** Baseline, surgical, and early postoperative radiographic predictors of unfavorable postoperative course.

Predictor	Univariable OR (95% CI)	*p* Value	Multivariable OR (95% CI)	*p* Value
Age, per year	1.05 (0.99–1.10)	0.082	1.05 (0.99–1.12)	0.085
Female sex (vs. male)	1.13 (0.45–2.81)	0.797	—	—
BMI, per kg/m^2^	1.11 (0.98–1.25)	0.097	—	—
ASA score, per class	1.24 (0.62–2.45)	0.544	—	—
Osteoporosis (vs. none)	2.98 (1.31–6.80)	0.009	3.97 (1.51–10.43)	0.005
Smoking (vs. non-smoker)	0.73 (0.31–1.70)	0.462	—	—
Fusion levels, per level	1.44 (1.17–1.77)	<0.001	1.49 (1.20–1.86)	<0.001
Pelvic fixation (vs. none)	0.74 (0.26–2.12)	0.579	—	—
Osteotomy use (vs. none)	0.66 (0.30–1.47)	0.310	—	—
Accessory/multi-rod construct (vs. 2-rod)	0.96 (0.38–2.40)	0.924	—	—
Preoperative PI–LL mismatch, per 10°	1.71 (1.12–2.62)	0.013	—	—
Preoperative SVA, per 10 mm	1.11 (0.97–1.26)	0.118	—	—
Immediate postoperative PI–LL mismatch, per 10°	1.52 (1.06–2.19)	0.023	1.58 (1.02–2.46)	0.040
Immediate residual sagittal imbalance (vs. none)	1.71 (0.75–3.90)	0.206	—	—

Notes: The outcome variable was unfavorable postoperative course. Revision/reoperation endpoint, late/mechanical complication, and deep infection were not included as predictors in this model because they were part of, or closely linked to, the composite outcome definition. The multivariable model included age, osteoporosis, number of fused levels, and immediate postoperative PI–LL mismatch to reduce overfitting. Preoperative PI–LL mismatch was not entered together with immediate postoperative PI–LL mismatch because of collinearity and the clinical priority of residual postoperative alignment. ORs for PI–LL mismatch are reported per 10° increase, and ORs for SVA are reported per 10 mm increase. Reference categories were male sex, no osteoporosis, non-smoker, no pelvic fixation, no osteotomy, standard two-rod construct, and no residual sagittal imbalance. ASA, American Society of Anesthesiologists; BMI, body mass index; CI, confidence interval; OR, odds ratio; PI–LL, pelvic incidence–lumbar lordosis mismatch; SVA, sagittal vertical axis.

## Data Availability

The data presented in this study are available from the corresponding author upon reasonable request, subject to institutional and ethical restrictions.

## Supplementary Materials

The following supporting information can be downloaded at https://www.mdpi.com/article/10.3390/jcm15135280/s1: Table S1: Correlation between immediate radiographic correction and clinical improvement. Table S2: Comparison of patients with and without available final ODI data. Table S3: Center-based comparison of baseline, operative, radiographic, and clinical variables.

## Abbreviations

The following abbreviations are used in this manuscript:

ASA	American Society of Anesthesiologists
BMI	body mass index
CI	confidence interval
CSF	cerebrospinal fluid
CT	computed tomography
DLS	degenerative lumbar scoliosis
HRQOL	health-related quality of life
MCID	minimum clinically important difference
ODI	Oswestry Disability Index
OR	odds ratio
PI–LL	pelvic incidence–lumbar lordosis
PJF	proximal junctional failure
PJK	proximal junctional kyphosis
PROMs	patient-reported outcome measures
PT	pelvic tilt
SRS	Scoliosis Research Society
SRS-22	Scoliosis Research Society-22
STROBE	Strengthening the Reporting of Observational Studies in Epidemiology
SVA	sagittal vertical axis
TLIF	transforaminal lumbar interbody fusion
TPA	T1 pelvic angle
UIV	upper instrumented vertebra
VAS	visual analog scale
