# Supplementary material for: Spinopelvic Realignment and Clinical Outcomes After Surgical Management of Adult Degenerative Lumbar Deformity: A Multicenter Retrospective Cohort Study
Source: J Clin Med. 2026 Jul 6;15(13):5280. doi: 10.3390/jcm15135280 (PMC13363233; doi:10.3390/jcm15135280)
Supplement: Supplementary file 1 [file jcm-15-05280-s001.zip › jcm-4358183-supplementary.pdf]

Supplementary Table S1. Correlation between immediate radiographic correction and clinical improvement

Radiographic correction parameter	ODI improvement (n = 95)	VAS back improvement (n = 98)	VAS leg improvement (n = 98)	SRS-22 total improvement (n = 93)
**Cobb reduction**	0.19 (0.063)	0.09 (0.399)	-0.09 (0.380)	0.06 (0.555)
**LL increase**	0.05 (0.644)	**0.26 (0.010)**	-0.10 (0.329)	0.19 (0.075)
**PI–LL reduction**	0.09 (0.382)	**0.26 (0.009)**	-0.08 (0.406)	0.19 (0.072)
**SVA reduction**	0.13 (0.206)	**0.54 (<0.001)**	-0.01 (0.946)	-0.08 (0.469)
**TPA reduction**	-0.06 (0.575)	0.08 (0.446)	-0.03 (0.737)	0.11 (0.273)

Notes. Values are Spearman's rank correlation coefficients with p values in parentheses; Spearman's test was used because several radiographic correction and patient-reported outcome variables were not normally distributed.

Radiographic correction was calculated as preoperative minus immediate postoperative value for Cobb angle, PI–LL mismatch, SVA, and TPA, and as immediate postoperative minus preoperative value for LL.

Clinical improvement was calculated as preoperative minus final follow-up value for ODI and VAS scores, and as final follow-up minus preoperative value for SRS-22 total score.

Analyses used available case pairwise data for each patient-reported outcome measure. $p < 0.05$ was considered statistically significant.

Abbreviations: LL, lumbar lordosis; ODI, Oswestry Disability Index; PI–LL, pelvic incidence–lumbar lordosis mismatch; SRS-22, Scoliosis Research Society-22; SVA, sagittal vertical axis; TPA, T1 pelvic angle; VAS, visual analog scale.

Supplementary Table S2. Comparison of patients with and without available final ODI data

Variable	Final ODI available (n = 95)	Final ODI unavailable (n = 18)	p value
**Baseline and surgical variables**			
Age, years	63.9 ± 7.8	61.1 ± 7.7	0.160
BMI, kg/m²	26.6 ± 3.3	26.4 ± 3.2	0.777
Fusion levels	7.5 ± 2.3	7.2 ± 2.2	0.785
Follow-up duration, months	31.5 ± 12.8	28.4 ± 13.6	0.407
Preoperative ODI	57.8 ± 12.6	55.6 ± 10.0	0.401
Preoperative PI–LL mismatch, °	22.4 ± 10.2	18.3 ± 8.4	0.079
Preoperative SVA, mm	60.6 ± 30.8	49.8 ± 33.8	0.224
Female sex	70 (73.7)	16 (88.9)	0.233
ASA score ≥ III	27 (28.4)	5 (27.8)	0.956
Osteoporosis	32 (33.7)	4 (22.2)	0.417
**Postoperative course and complications**			
Any postoperative complication	42 (44.2)	6 (33.3)	0.392
Late/mechanical complication	29 (30.5)	4 (22.2)	0.581
Revision/reoperation endpoint	27 (28.4)	0 (0.0)	**0.006**
Unfavorable postoperative course	40 (42.1)	0 (0.0)	**<0.001**

Notes. Values are presented as mean ± standard deviation or n (%), unless otherwise indicated.

Continuous variables were compared using Welch t-test or Mann–Whitney U test according to distributional assumptions; categorical variables were compared using chi-square or Fisher's exact test, with Fisher's exact test used when expected cell counts were small or zero.

Missing final ODI values reflected incomplete PROM documentation rather than absence of clinical or radiographic follow-up.

Abbreviations: ASA, American Society of Anesthesiologists; BMI, body mass index; ODI, Oswestry Disability Index; PI–LL, pelvic incidence–lumbar lordosis mismatch; PROM, patient-reported outcome measure; SVA, sagittal vertical axis.

Supplementary Table S3. Center-based comparison of baseline, operative, radiographic, and clinical variables

Variable	Center 1 (n = 36)	Center 2 (n = 38)	Center 3 (n = 39)	p value
Age, years	63.8 ± 7.7	62.9 ± 8.2	63.7 ± 7.8	0.701
Female sex	28 (77.8)	26 (68.4)	32 (82.1)	0.386
BMI, kg/m²	27.4 ± 3.5	26.0 ± 3.1	26.4 ± 3.4	0.087
ASA ≥ III	10 (27.8)	9 (23.7)	13 (33.3)	0.656
Osteoporosis	14 (38.9)	12 (31.6)	10 (25.6)	0.466
Follow-up duration, months	32.4 ± 12.6	30.6 ± 11.6	30.3 ± 14.6	0.687
Fusion levels	7.4 ± 2.5	7.3 ± 2.3	7.7 ± 2.2	0.635
Distal fixation to S1/pelvis	31 (86.1)	31 (81.6)	34 (87.2)	0.801
Pelvic fixation	4 (11.1)	5 (13.2)	11 (28.2)	0.129
TLIF	31 (86.1)	36 (94.7)	36 (92.3)	0.416
Operative time, min	363.5 ± 59.3	351.4 ± 51.0	366.1 ± 44.6	0.577
Estimated blood loss, mL	1034.3 ± 264.4	978.9 ± 308.2	984.7 ± 265.7	0.642
Preoperative ODI	57.0 ± 11.8	54.0 ± 12.5	61.3 ± 11.5	**0.041**
Preoperative PI–LL mismatch, °	20.5 ± 9.6	21.3 ± 9.1	23.3 ± 11.2	0.551
Preoperative SVA, mm	49.9 ± 31.2	62.7 ± 31.4	63.4 ± 30.6	0.103
Immediate PI–LL mismatch, °	9.5 ± 11.4	10.1 ± 10.7	10.6 ± 12.6	0.874
Immediate SVA, mm	25.1 ± 37.2	37.7 ± 32.5	34.3 ± 37.9	0.262
ODI improvement	21.3 ± 14.1	20.7 ± 13.2	27.2 ± 11.8	0.096
Any postoperative complication	14 (38.9)	14 (36.8)	20 (51.3)	0.412
Late/mechanical complication	12 (33.3)	7 (18.4)	14 (35.9)	0.192
Revision/reoperation endpoint	11 (30.6)	6 (15.8)	10 (25.6)	0.329
Unfavorable postoperative course	11 (30.6)	17 (44.7)	12 (30.8)	0.353

Values are presented as mean ± standard deviation or n (%).

Continuous variables were compared using one-way ANOVA or the Kruskal–Wallis test according to distributional assumptions. Binary categorical variables were compared across centers using the Fisher–Freeman–Halton exact test. Significant p values are shown in bold.

Diagnostic and operative categories are not mutually exclusive where applicable.

Abbreviations: ASA, American Society of Anesthesiologists; BMI, body mass index; ODI, Oswestry Disability Index; PI–LL, pelvic incidence–lumbar lordosis mismatch; SVA, sagittal vertical axis; TLIF, transforaminal lumbar interbody fusion.
